# Airway obstruction produces widespread sympathoexcitation: role of hypoxia, carotid chemoreceptors, and NTS neurotransmission

**DOI:** 10.14814/phy2.13536

**Published:** 2018-02-01

**Authors:** Caroline B. Ferreira, Sergio L. Cravo, Sean D. Stocker

**Affiliations:** ^1^ Department of Physiology Federal University of São Paulo São Paulo São Paulo Brazil; ^2^ Department of Medicine Division of Renal‐Electrolyte University of Pittsburgh School of Medicine Pittsburgh Pennsylvania; ^3^ University of Pittsburgh Hypertension Center University of Pittsburgh School of Medicine Pittsburgh Pennsylvania

**Keywords:** Apnea, carotid chemoreceptors, NTS, sympathetic nerve activity

## Abstract

Obstructive sleep apnea (OSA) is the most common respiratory disturbance of sleep and is closely associated to cardiovascular diseases. In humans, apnea increases respiratory effort and elevates muscle sympathetic nerve activity (SNA), but the primary stimulus for the SNA activation has not been identified. We recently developed a model of apnea in rodents using acute airway obstruction. In this study, we employed this model to test whether the elevation in SNA was mediated by hypoxia, carotid chemoreceptors, or neurotransmission in the nucleus tractus solitarius (NTS). In anesthetized, male Sprague–Dawley rats, airway obstruction (20s) increased phrenic nerve activity (PNA), arterial blood pressure (ABP), and lumbar, renal, and splanchnic SNA. The changes in SNA were similar across all three sympathetic nerves. Inactivation of chemoreceptors by hyperoxia (100% O_2_) or surgical denervation of carotid chemoreceptors attenuated, but did not eliminate, the changes in SNA and ABP produced by airway obstruction. To interrupt afferent information from carotid chemoreceptor and extracarotid afferents to the hindbrain, airway obstruction was performed before and after NTS microinjection of the GABA_A_ agonist muscimol or a cocktail of NMDA and non‐NMDA antagonists. Inhibition of NTS neurons or blockade of glutamatergic receptors attenuated the increase in lumbar SNA, splanchnic SNA, renal SNA, and PNA. Collectively, these findings suggest that PNA and SNA responses induced by airway obstruction depend, in part, on chemoreceptors afferents and glutamatergic neurotransmission in the NTS.

## Introduction

Obstructive sleep apnea (OSA) is characterized by partial or complete cessation of breathing with consequent hypoxemia, hypercapnia, and gradual increase in respiratory effort (Dempsey et al. 2010). An episode of OSA in humans progressively increases muscle sympathetic nerve activity (SNA) and peaks at the end of apnea (Leuenberger et al. 1995; Somers et al. 1995). Prior studies indicate that hypoxia triggers the SNA response to OSA or acute apnea (Leuenberger et al. 1995; O'Donnell et al. 1996). For example, the muscle SNA response to apneas in humans was significantly attenuated during hyperoxia (breathing 100% oxygen) versus room air (Leuenberger et al. 1995). In addition, renal sympathoexcitatory response to apneas in anesthetized cats was attenuated during hyperoxia versus room‐air (O'Donnell et al. 1996). The primary set of chemoreceptors is located in the bifurcation of the common carotid arteries. These cells can detect changes in po_2_ and in pco_2_ and relay afferent information through the glossopharyngeal nerve to release glutamate onto neurons of the nucleus tractus solitarius (NTS) (Kumar and Prabhakar 2012). Central integration of these afferents signals produces subsequent changes in phrenic nerve activity (PNA) and respiratory function as well as sympathetic and cardiovascular adjustments. The above‐mentioned studies highlight the contribution of hypoxia as an important stimulus (Leuenberger et al. 1995; O'Donnell et al. 1996). However, the inactivation of carotid chemoreceptors by hyperoxia partially attenuated the SNA responses in both humans and experimental models. This observation suggests that another stimulus aside from hypoxia causes sympathoexcitation. Interestingly, carotid body denervation (CBD) eliminates both phrenic nerve activity (PNA) and SNA responses to hypoxia (Koshiya and Guyenet 1996). Thus, the purpose of this study was to test whether airway obstruction activates carotid body afferents to increase SNA and arterial blood pressure (ABP).

We recently developed a model of obstructive sleep apnea produced by inflation of a trachea balloon (Schoorlemmer et al. 2011). A 15 sec balloon inflation decreases arterial O_2_ saturation ~20%, increases CO_2_ and respiratory effort, and produces a pressor response (Schoorlemmer et al. 2011). Carotid body denervation (CBD) attenuates the cardiovascular responses to airway obstruction in nonanesthetized rats (Angheben et al. 2014). Furthermore, CBD did not reduce the neuronal activation evoked by obstructive apnea in ventrolateral medulla of regions involved in the respiratory and autonomic control (Ferreira et al. 2015). Collectively, the above observations suggest the respiratory and sympathetic changes induced by upper airway obstruction may be partially mediated by carotid chemoreceptors.

This study directly tested the above hypothesis through simultaneous recordings of phrenic nerve activity (PNA), lumbar SNA, renal SNA, and splanchnic SNA during airway obstruction in anesthetized rats. Airway obstruction was induced by clamping a tracheal tube. This procedure mimics apnea in humans as it produces gradual changes in blood oxygen and CO_2_ and increases respiratory effort against a closed upper airway that affect thoracic pressure, hemodynamics, and airway sensors. The contribution of chemoreceptor function was tested by three ways: (1) inactivation of chemoreceptors by hyperoxia (100% O_2_), (2) surgical CBD, and (3) inhibition of NTS neurons or blockade of glutamatergic receptors in the NTS. The simultaneous recording of multiple sympathetic nerves also provided additional insight into whether apnea produced differential changes in SNA.

## Materials and Methods

All experimental procedures were approved by the Institutional Animal Care and Use Committee at the Pennsylvania State College of Medicine and based in the *National Institutes of Health Guide for the Care and Use of Laboratory Animals*. Male Sprague–Dawley rats (250–350 g, Charles Rives Laboratories) were kept in a 12:12 h light–dark cycle room with controlled temperature (23 ± 1°C). The rats had free access to deionized water and standard chow (Harlan Teklad Global Diet 2018).

### General procedures

Initially, rats were anesthetized with isoflurane (2–3% in 100% O_2_) and instrumented with femoral arterial and venous catheters to measure ABP and administer drugs, respectively. In some rats, a brachial arterial catheter was implanted to collect blood samples. The trachea was cannulated using a metal tube attached to a plastic tube and the rats were spontaneously breathing. The anesthesia was then replaced by urethane (1.2 g/kg, IV) administered over 10 min. Oxygen saturation was monitored by an oximeter (mouseOx; Starr Life Science), and end‐tidal CO_2_ was monitored by a capnometer (Microcapstar‐100; CWE). Body temperature was measured rectally (Sable Systems) and maintained at 37 ± 0.5°C by a water‐circulating pad. The rats were prepared for simultaneous recording of lumbar, renal, and splanchnic SNA through a ventral midline and retroperitoneal approach as described previously (Simmonds et al. 2014; Stocker and Gordon 2015). The animals were placed in a stereotaxic frame, and the phrenic nerve was accessed after left shoulder retraction using a dorsolateral approach. All nerves were placed on a bipolar stainless‐steel electrode and insulated with a silicon elastomer (Kwik‐Sil; World Precision Instruments). The raw signals were filtered (100–1000 Hz), amplified (×10,000), digitized (sampling rate 2 kHz), rectified and integrated (*τ* = 20 msec and 50 msec for SNA and PNA, respectively) using a Micro1401 and Spike2 software (Cambridge Electronics Design).

### Experimental design

#### Experiment 1

Initial experiments investigated the extent by which airway obstruction produced regional changes in SNA across lumbar, renal, and splanchnic nerves. First, the airway obstructions were induced by clamping the tracheal tube for 20 s independently of respiratory cycle in rats (n=14, ≈10 apneas) breathing room air. Airway obstructions trials were repeated every 2 min. In a subset of rats, arterial blood samples (70*μ*L) were collected from the brachial arterial catheter, before and between the last seconds of obstruction to analyze pH, pco_2_, po_2_, and arterial saturation using an I‐STAT CG8 +  cartridge. Blood volume was replaced with isotonic saline.

#### Experiment 2

To test the contribution of hypoxia to respiratory, sympathetic, and cardiovascular changes induced by airway obstructions, the obstructions were repeated in animals (see experiment 1) breathing 100% oxygen for 2 min prior to the apnea. Again, arterial blood samples were collected to analyze pH, pco_2_, po_2_, and arterial saturation.

#### Experiment 3

To test the contribution of the peripheral chemoreceptor afferents to the respiratory, sympathetic, and cardiovascular changes induced by airway obstructions, bilateral CBD was performed surgically in a separate group of rats (*n* = 9) by section of the carotid body artery and carotid sinus nerve branches. First, the denervation was assessed by changes in PNA and SNA to IV injection of NaCN (40 *μ*g/100 *μ*L). Rats were breathing freely 100% of oxygen to maintain a stable preparation, and the airway obstructions were performed as described above. To confirm that cardiovascular responses to airway obstruction depend on the autonomic nervous system, airway obstructions were repeated after treatment with the ganglionic blocker (chlorisondamine, 5 mg/kg IV).

#### Experiment 4

A final set of experiments assessed the contribution of NTS neurotransmission to changes in PNA, SNA, and ABP during airway obstruction, whereas rats were breathing 100% of oxygen. After animals were placed into a stereotaxic frame, a small craniotomy was performed to expose the dorsal surface of the hindbrain and area postrema. A glass pipette (30 *μ*m OD) was inserted in the NTS with incisor bar positioned 11 mm below the interaural line using the following coordinates: 0.3–0.5 mm rostral, 1 mm lateral, and 0.6–1 mm ventral of the *calamus scriptorius*. These coordinates target a large extension of the NTS, including dorsolateral, ventrolateral, mediolateral, and commissural subnuclei (Gourine et al., 2008). Microinjections (50 nL over 5 sec) were performed bilaterally separated by 1 min using a picopump and eye reticle. All apnea‐induced responses were tested before and every 5 min after NTS injection of aCSF (50 nL per side), a cocktail of NMDA antagonist DL‐2‐amino‐5‐phosphonopentanoic acid (AP5, 4 mmol/L), and non‐NMDA antagonist 6‐cyano‐7‐nitroquinoxaline‐2,3‐dione (CNQX, 2 mmol/L), or the GABA_A_ agonist muscimol (2 mmol/L). Muscimol was injected at the end of experiments. To verify the efficacy of NTS injections, PNA and SNA responses to NaCN (40 *μ*g/100 *μ*L, IV) and phenylephrine (4 *μ*g/kg, IV) were tested before and 5–10 min after NTS injections. At the end, rats were perfused transcardially with heparinized saline followed by 4% paraformaldehyde. The brain was sectioned at 100 *μ*m using a vibratome. The injection site was identified by the addition of fluorescent bead (0.2%) to the drug solution.

### Statistical analysis

The background noise was determined at the end of experiments after euthanasia with a high dose of urethane or signal voltage between bursts. All data represent viable nerve recordings assessed by a 2:1 signal‐to‐noise ratio as previously described (Stocker and Gordon, 2015). SNA and PNA was calculated by subtracting background noise from the integrated value and then normalized to a baseline value to 100% for each animal. Frequency of phrenic discharge was assessed by identifying the inspiratory peaks and calculating the frequency of burst per min. Data are reported as means ± SEM and peak changes (1s). HR values were averaged over the last 3 sec of the apnea. Graphs and analyses were done using GraphPad Prism version 5. The Student's t test and one‐way analysis of variance were used as appropriate. Significance level was set at *P* ≤ 0.05.

## Results

### Experiment 1. Airway obstruction increases in lumbar, renal, and splanchnic SNA

**Figure 1:**
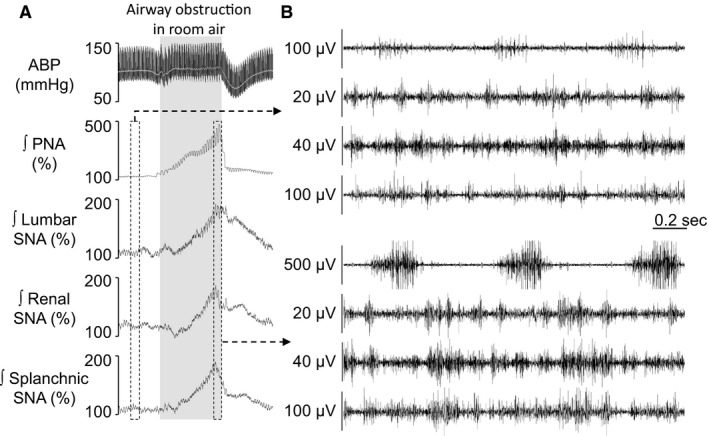
Airway obstruction induces similar sympathetic activation across lumbar, renal, and splanchnic nerves. A: Examples of ABP, mean ABP (gray line), rectified and integrated (ʃ) phrenic, lumbar, renal, and splanchnic nerve activity, in response to 20 sec of upper airway obstruction. B: Raw phrenic, lumbar, renal, and splanchnic nerve activities at rest (top) and 2 sec of end apnea (bottom).

The first purpose of this study was to assess whether airway obstruction induces regional changes in SNA. Baseline mean ABP, heart rate (HR), frequency of phrenic nerve activity (PNA), and end‐tidal CO_2_ are reported in Table 1. Figure 1A illustrates a typical example of changes in ABP, PNA, and SNA across lumbar, renal, and splanchnic nerves during 20 sec of upper airway obstruction. The obstruction promptly increased PNA within 1–2 sec. Lumbar SNA, renal SNA, splanchnic SNA, and ABP increased at 4–5 sec and reached a peak in the last second of obstruction. The magnitude of the sympathoexcitation induced by upper airway obstruction was not statistically different across various end‐organs (ΔlumbarSNA: 117 ± 13% vs. ΔrenalSNA: 114 ± 15% vs. ΔsplanchnicSNA: 123 ± 12%, *P* > 0.337).

**Table 1:** Means arterial blood pressure (ABP), heart rate (HR), frequency of phrenic nerve activity, and end‐tidal CO_2_ at rest

	Chemo intact		CBD	
	21% O_2_	100% O_2_	100% O_2_	100% O_2_ after ganglionic blocker
Mean ABP (mmHg)	90 ± 2	96 ± 2*	98 ± 2	49 ± 3†
HR (bpm)	409 ± 14	422 ± 13	434 ± 14	388 ± 6†
Frequency PNA (burst/min)	102 ± 7	96 ± 5	104 ± 7	100 ± 6
End‐tidal CO_2_ (%)	5 ± 0.2	5 ± 0.2	5.2 ± 0.3	4.6 ± 0.4

Values are mean ± SEM. *P < 0.05, versus chemo intact breathing 21% O_2_ and †*P* < 0.05 versus carotid body denervated (CBD).

### Experiment 2. Hyperoxia attenuates cardiorespiratory and sympathetic responses to airway obstruction

**Figure 2:**
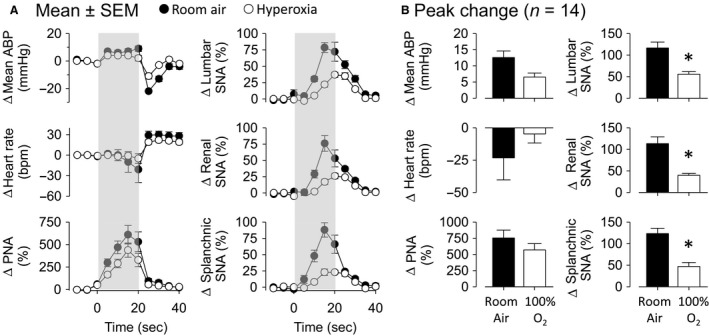
Hyperoxia attenuated cardiovascular and sympathetic responses induced by airway obstruction. A: Changes in mean ABP, heart rate (HR), phrenic nerve activity (PNA), lumbar, renal, and splanchnic SNA in rats subjected to 20 sec of airway obstruction (gray‐shaded area) in room air or hyperoxia. B: Peak changes from data presented in A. *P < 0.05, between room air and hyperoxia.

To test the contribution of hypoxia, the airway obstructions were induced, whereas animals were freely breathing 100% O_2_. Hyperoxia slightly increased baseline mean ABP and HR when compared with room air (21% O_2_) (Table 1). Figure 2A illustrates the changes in cardiovascular, respiratory, and sympathetic responses as a function of time to airway obstruction during room air or hyperoxia. Figure 2B illustrates peak changes in mean ABP, HR, PNA, and SNA across lumbar, renal, and splanchnic nerves. Hyperoxia attenuated the peak increase in mean ABP, lumbar SNA, renal SNA, and splanchnic SNA induced by airway obstruction. Hyperoxia did not statistically change the increase in PNA and HR response. All variables returned to baseline within 10–15 sec after the end of the obstruction regardless of room‐air or hyperoxia conditions. HR returned to baseline after 30 sec.

In six rats, arterial blood samples were analyzed to assess changes in blood gases evoked by airway obstruction during room air and hyperoxia. As expected, 20 sec of airway obstruction reduced po_2_, arterial O_2_ saturation, and pH but increased pco_2_ during room air and hyperoxia (Table 2). Note that, po_2_ and arterial O_2_ saturation values at the end of airway obstruction during hyperoxia approached those values obtained when animals were breathing room air at rest. Thus, hypoxia was avoided when the airway obstructions were performed during hyperoxia (Table 2).

**Table 2:** Partial pressure of oxygen (po_2_), oxygen saturation (Sat O_2_), partial pressure of carbon dioxide (pco_2_) e pH at basal, and final seconds of airway obstruction in rats breathing air way (21% O_2_) or hyperoxia (100% O_2_)

	po_2_ (mmHg)		Sat O_2_ (%)		pco_2_ (mmHg)		pH	
F_i_O_2_	Basal	Final	Basal	Final	Basal	Final	Basal	Final
21% O_2_	72 ± 2	45 ± 1*	94 ± 0.5	78 ± 3*	40 ± 1	45 ± 3*	7.41 ± 0	7.39 ± 2*
100% O_2_	240 ± 33†	71 ± 5*, †	99 ± 0.2†	93 ± 1*, †	45 ± 1†	53 ± 3*, †	7.39 ± 0†	7.32 ± 0*, †

Values are mean ± SEM. *P < 0.05 versus basal and ^†^ *P* < 0.05 versus breathing 21% O_2_.

### Experiment 3. Carotid chemoreceptors partially contribute to PNA and sympathoexcitatory responses during airway obstruction

To investigate the contribution of carotid chemoreceptors afferents, the cardiorespiratory and sympathetic responses to airway obstruction were tested in a separate group of rats after carotid body denervation (CBD) but breathing 100% O_2_. Baseline mean ABP, heart rate, frequency of PNA, and end‐tidal CO_2_ in a CBD rats are showed in Table 1.

**Figure 3:**
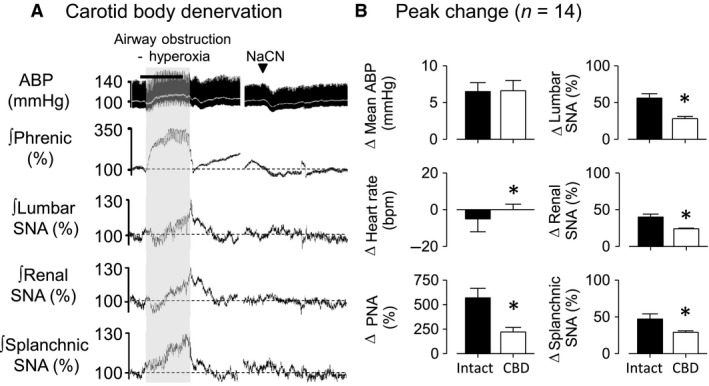
Acute carotid body denervation did not prevent cardiorespiratory and sympathetic responses induced by airway obstruction. A: Examples of ABP, rectified and integrated (ʃ) phrenic, lumbar, renal, and splanchnic nerve activity in response to airway obstruction (20 sec, gray‐shaded area) while breathing hyperoxia and NaCN IV injection. B: Changes in mean ABP, heart rate (HR), phrenic nerve activity (PNA), lumbar, renal, and splanchnic SNA in chemo intact rats versus carotid body denervated (CBD) rats subjected in response to airway obstruction in hyperoxia. *P < 0.05, between chemo intact and CBD.

Figure 3A illustrates an example of cardiovascular, respiratory, and sympathetic responses to airway obstruction in a CBD rat. Airway obstruction after CBD increased ABP, PNA, and SNA across lumbar, renal, and splanchnic nerves (Fig. 3A). However, CBD did reduce the magnitude of the changes in HR, PNA, lumbar SNA, renal SNA, and splanchnic SNA (Fig. 3B). There were no differences in the pressor response between intact versus CBD rats. CBD was verified by the absence of changes in ABP, PNA, and SNA in response to NaCN (Fig. 3A).

To verify whether pressor response to airway obstruction in CBD rats could be mediated by autonomic nervous system, obstructions were tested after administration of the ganglionic blocker. As expected, chlorisondamine promptly reduced SNA, mean ABP, and HR from baseline values with no change in PNA frequency (Table 1). Ganglionic blockade eliminated the pressor (BEFORE: Δmean ABP: 7 ± 1 mmHg vs. AFTER: 1 ± 1 mmHg; *P* < 0.05) and changed HR response (BEFORE: ΔHR: 0 ± 3 bpm vs. AFTER: ‐20 ± 8 bpm; *P* < 0.05) induced by airway obstruction in CBD rats. As expected, ganglionic blockade did not affect changes in PNA (BEFORE: ΔPNA: 221 ± 47% vs. AFTER: 251 ± 56%; *P* > 0.05).

### Experiment 4. Interruption of NTS neurotransmission attenuates PNA, SNA, and cardiovascular responses to upper airway obstruction

**Figure 4:**
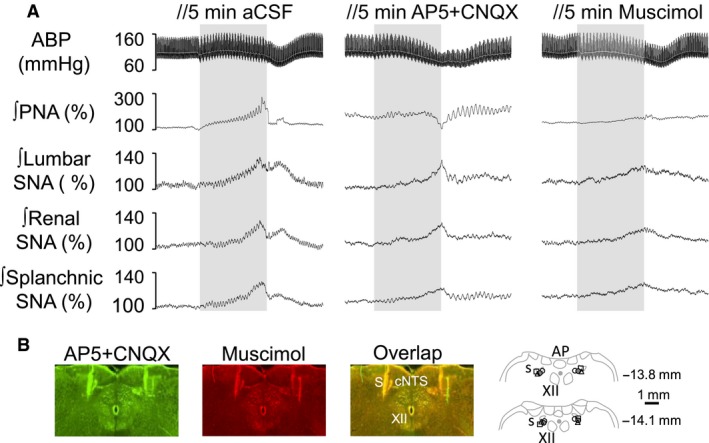
NTS neurotransmission contributes in part to airway obstruction‐induced responses. A: Examples of ABP, mean ABP (gray line), rectified and integrated (ʃ) phrenic, lumbar, renal, and splanchnic nerve activity in response to 20 sec of airway obstruction (gray bar) in hyperoxia, tested after 5 min injection of artificial cerebrospinal fluid (aCSF), AP5 + CNQX cocktail or muscimol. B: Digital images of the NTS injections sites for AP5 + CNQX (green), muscimol (red), or combined AP5 + CNQX plus muscimol images (yellow). Schematic illustrations demonstrating injection sites at caudal level of NTS. The numbers at the left of each coronal sections corresponding the distance from the bregma according to the rat brain atlas of Paxinos and Watson (1997). AP: area postrema, cc: central canal, commNTS: commissural nucleus of the tract solitary, sol: solitary tract, XII: hypoglossal nerve.

**Figure 5:**
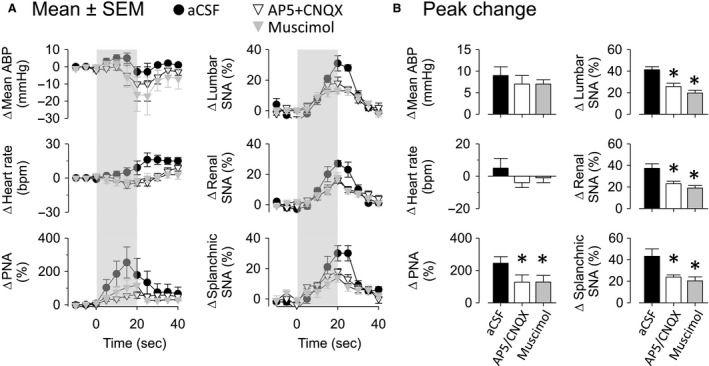
Cardiorespiratory and sympathetic responses to airway obstruction were reduced after interruption of NTS neurotransmission. A: Changes in mean arterial blood pressure (ABP), heart rate (HR), phrenic nerve activity (PNA), lumbar, renal, and splanchnic sympathetic nerve activity (SNA), after 5 minutes of drug injections into NTS in the same group of rats. B: Changes in data presented in A. aCSF: artificial cerebral spinal fluid. *P ≤ 0.05, versus aCSF.

Airway obstruction like obstructive apnea, results in hypoxemia, hypercapnia, intense respiratory effort, sympathetic activation, and pressor responses. It is likely that those responses might modify the activities of several sensors including chemoreceptors, baroreceptors, and pulmonary receptors. Since is well‐known that the afferents from those receptors terminate in the NTS, we employed a final approach and investigated the role of NTS neurotransmission in the cardiorespiratory and sympathetic responses produced by airway obstruction in rats breathing 100% of oxygen. The responses were tested after injection of aCSF, a cocktail of NMDA plus non‐NMDA receptor antagonists to block glutamatergic receptors, or a GABA_A_ agonist to inhibit NTS neurons. Figure 4 illustrates examples of the ABP, PNA, and SNA during airway obstruction at 5–10 min after injection of aCSF, AP5 + CNQX cocktail or muscimol. Summary data are presented in Figure 5. Bilateral microinjection of aCSF did not alter baseline values of mean ABP, HR, PNA, or SNA to lumbar, renal, and splanchnic nerves (Table 3). Airway obstruction after aCSF injection increased ABP, PNA, and SNA. Bilateral injection of AP5 + CNQX cocktail promptly increased basal mean ABP (98 ± 3 mmHg to 111 ± 6 mmHg; *P* < 0.05), lumbar SNA (100 ± 0% to 122 ± 3%; *P* < 0.05), renal SNA (100 ± 0% to 119 ± 5%; *P* < 0.05), and splanchnic SNA (100 ± 0% to 123 ± 4%; *P* < 0.05) but reduced PNA frequency (100 ± 13 burst/min to 60 ± 3 burst/min; *P* < 0.05). HR did not change (Table 3). Blockade of NMDA/non‐NMDA receptors in the NTS reduced PNA and SNA responses to airway obstruction (Figs. 4 and 5). Interestingly, airway obstruction after injection of AP5 + CNQX produced a biphasic mean ABP response. Mean ABP increased within 4–5 sec of obstruction but then decreased significantly below baseline values. However, the peak increase in mean ABP was not different between rats injected with aCSF vs. AP5 + CNQX (Fig. 5).

**Table 3:** Arterial blood pressure, heart rate, phrenic nerve frequency, and end‐expiratory co_2_ at baseline and after 5–10 min of drug injections

	Experiment 4			
	Before injections	aCSF	AP5 + CNQX	Muscimol
Mean ABP (mmHg)	98 ± 3	94 ± 5	111 ± 6*	116 ± 8*
HR (bpm)	398 ± 19	434 ± 19	395 ± 24	434 ± 17
Frequency PNA (burst/min)	100 ± 13	100 ± 5	60 ± 3*	66 ± 7*
Lumbar SNA (%)	100 ± 0	104 ± 2	122 ± 3*	122 ± 10*
Renal SNA (%)	100 ± 0	98 ± 7	119 ± 5*	125 ± 8*
Splanchnic SNA (%)	100 ± 0	104 ± 5	123 ± 4*	122 ± 8*
End‐tidal co_2_ (%)	5 ± 0.2	5.6 ± 0.3	5.8 ± 0.3	5.9 ± 0.3

Values are mean ± SEM. *P < 0.05, different from before injections.

Injection of muscimol into NTS neurons promptly increased basal mean ABP (98 ± 3 mmHg to 116 ± 8 mmHg; *P* < 0.05), lumbar SNA (100 ± 0% to 122 ± 10%; *P* < 0.05), renal SNA (100 ± 0% to 125 ± 8%; *P* < 0.05), and splanchnic SNA (100 ± 0% to 122 ± 8%; *P* < 0.05) but reduced PNA frequency (100 ± 13 burst/min to 66 ± 7 burst/min; *P* < 0.05) (Table 3). Injection of muscimol also attenuated the PNA and SNA responses and produced a biphasic ABP response to airway obstruction (Figs. 4 and 5).

Figure 4B provides digital images and schematic illustration of NTS injection sites. Note that the injections occurred mainly around the solitary tract. As demonstrated by combined digital images (yellow), the injections of muscimol (red) occurred at the same level of AP5 + CNQX injection (green).

**Figure 6:**
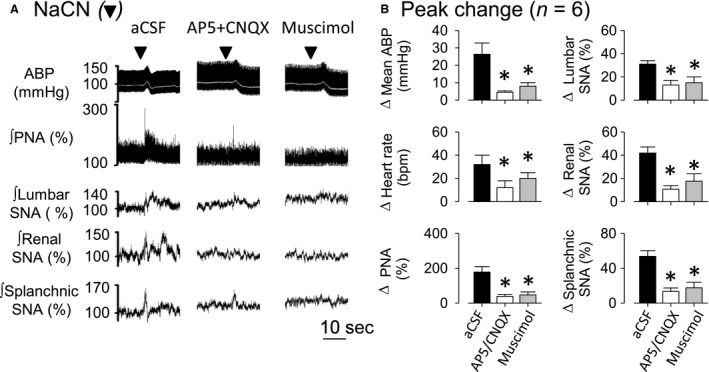
Chemoreflex was strongly affected by interruption of NTS neurotransmission. A: Examples of ABP, mean ABP (gray line), rectified and integrated (ʃ) phrenic, lumbar, renal, and splanchnic sympathetic nerve activity (SNA) in response to NaCN IV tested after injection of artificial cerebral spinal fluid (aCSF), AP5 + CNQX cocktail or muscimol into NTS. Black arrows represent time of NaCN IV injection. B: Changes of mean ABP, heart rate (HR), phrenic nerve activity (PNA), lumbar, renal, and splanchnic SNA in response to NaCN tested after 10 min of drug microinjections into NTS. Values are means ± SEM. *P < 0.05, versus aCSF.

To confirm whether the above injections blocked afferent input from carotid chemoreceptors and baroreceptors, we evaluated the chemoreflex responses to NaCN and baroreflex responses to phenylephrine. Figure 6 illustrates an example of changes in mean ABP, PNA, and SNA to IV injection of NaCN at 5–10 min after bilateral NTS microinjection of aCSF, AP5 + CNQX cocktail or muscimol. Blockade of NMDA/non‐NMDA receptors or inhibition of NTS neurons largely reduced pressor response, PNA, and sympathetic activation to NaCN (Fig. 6). Furthermore, both treatments significantly attenuated the sympathoinhibitory baroreflex responses mediate to phenylephrine (Table 4).

**Table 4:** Changes in mean ABP, heart rate, lumbar SNA, renal SNA, and splanchnic SNA evoked by phenylephrine after 5‐10 min of pharmacological pretreatment into NTS

Treatment	Δ ABP (mmHg)	Δ HR (bpm)	Δ Lumbar SNA (%)	Δ Renal SNA (%)	Δ Splanchnic SNA (%)
aCSF	49 ± 4	−52 ± 11	−81 ± 6	−80 ± 3	−88 ± 5
AP5 + CNQX	43 ± 7	−71 ± 26	−17 ± 4*	−16 ± 3*	−16 ± 3*
Muscimol	43 ± 11	−78 ± 26	−26 ± 12*	−31 ± 15*	−35 ± 16*

Values are means ± SEM. *P < 0.05, different from aCSF injection.

## Discussion

The sympathoexcitatory response to apnea or OSA is triggered, at least partly, by hypoxia (Leuenberger et al. 1995; Somers et al. 1995; O'Donnell et al. 1996; Imadojemu et al. 2002). This study tested the hypothesis that apnea may activate carotid body afferents and NTS neurotransmission to alter PNA, SNA, and ABP. Herein, we provide several novel observations including: (1) airway obstruction evokes similar increases in SNA across lumbar, renal, and splanchnic nerves, (2) hyperoxia partially attenuates these sympathetic and cardiovascular responses, (3) absence of carotid chemoreceptors afferents by CBD attenuates PNA and SNA responses to airway obstruction, and (4) inhibition of NTS neurons or blockade of glutamate receptors attenuates respiratory and sympathoexcitatory responses to airway obstruction. Altogether, these observations suggest that the cardiovascular, respiratory, and sympathetic responses induced by airway obstruction partly depend on peripheral chemoreceptors and NTS neurotransmission.

### Sympathetic neural responses to upper airway obstruction

Previous studies have documented that OSA increases muscle SNA and ABP in humans (Leuenberger et al. 1995), whereas apnea increases renal SNA in anesthetized cats (O'Donnell et al. 1996). This study performed simultaneous sympathetic nerve recordings and provided the first evidence that airway obstruction increases SNA in lumbar, renal, and splanchnic nerves. These sympathoexcitatory responses are functionally significant as the ganglionic blocker chlorisondamine eliminated the pressor response to airway obstruction. In addition, these data suggest that OSA patients might have widespread sympathetic activation during an obstructive apnea. Despite large increases in SNA, ABP increased ~10 mmHg (room air). The relatively small impact on ABP may be attributed to a number of factors including changes in intrathoracic pressure, direct effects of hypoxia on the vasculature, and anesthesia. Sympathetic tone arises from the tonic drive of neurons in the rostral ventrolateral medulla (RVLM) to preganglionic neurons in the spinal cord (Guyenet 2006). RVLM neurons are lie immediately adjacent to the ventral respiratory column. Furthermore, changes in respiratory neurons activity can modify sympathetic neuronal activity in the brainstem (Haselton and Guyenet 1989; Spyer and Gourine 2009; Moraes et al. 2014). Future studies need to address whether changes in the activity of respiratory networks are necessary for the changes in SNA during airway obstruction.

### The effect of hypoxia and CBD on apnea‐induced responses

A major goal of this study was to investigate the contribution of hypoxia and carotid chemoreceptors afferents in the cardiorespiratory responses to airway obstruction. Carotid chemoreceptors promptly increase discharge frequency of the carotid sinus nerve when po_2_ falls below 60 mmHg (Kumar and Prabhakar 2012). Changes in po_2_ above 60 mmHg have a much smaller effect, and the basal activity of carotid sinus nerve when animals are breathing 100% O_2_ is very low (Vidruk et al. 2001). Administration of 100% of oxygen in subjects during obstructive apnea attenuated muscle SNA and pressor responses (Leuenberger et al. 1995; Imadojemu et al. 2002). Hyperoxia also reduces the renal sympathoexcitatory response to apnea in anesthetized cats (O'Donnell et al. 1996). In this study, airway obstruction during hyperoxia changed the po_2_ from 240 ± 33 to 72 ± 6 mmHg, and the nadir arterial oxygen saturation remained above 90%. Although, we did not record carotid sinus nerve activity, po_2_ remained above 70 mmHg which is unlikely to powerfully activate carotid sinus nerve (Vidruk et al. 2001). Hyperoxia attenuated, but did not eliminate, the SNA, HR, and ABP responses to airway obstruction. Therefore, these results suggest hypoxia partially contributes to the SNA and cardiovascular responses induced by obstructive apnea.

Airway obstruction also increased pco_2_. Therefore, the increase in CO_2_ may activate carotid body chemoreceptors to elevate discharge frequency of the carotid sinus nerve or directly activate central chemosensitive neurons (Moreira et al. 2006; Kumar and Prabhakar 2012). In a second set of experiments, airway obstruction was performed in CBD rats to directly investigate the contribution of carotid chemoreceptor afferents. CBD abolishes respiratory and sympathetic responses to hypoxia (Koshiya and Guyenet 1996; Takakura et al. 2006). In this study, CBD rats were denervated as NaCN did not produce changes in PNA, SNA, or mean ABP. However, the increase in PNA and sympathoexcitation induced by airway obstruction was partially attenuated. These results are consistent with previous report that CBD did not reduce Fos immunoreactivity in ventrolateral medulla of autonomic and respiratory regions (Ferreira et al. 2015) or the pressor response (Angheben et al. 2014) to airway obstruction. Collectively, these findings indicate that activation of carotid chemoreceptor afferents partially contribute to the sympathetic and cardiorespiratory changes during airway obstruction. These observations might have clinical relevance as OSA patients with normal or small oxygen desaturation may still exhibit elevated SNA independent of hypoxia or carotid chemoreceptor mechanisms.

So, how could the increase in CO_2_ concentration contribute to the cardiovascular, respiratory, and sympathetic responses induced by airway obstruction after CBD? The retrotrapezoid nuclei (RTN) are a well‐known central chemoreceptor located in the rostral area of medulla oblongata close to the ventral surface. Their neurons can increase the discharge frequency by increases in CO_2_ and detection of ions H^+^ in the extracellular fluid (Guyenet et al. 2016). In addition, astrocytes might also contribute to the chemosensitive response (Gourine et al. 2010). Previously, one study reported that sympathoexcitatory response evoked by hypercapnia occur by small changes in CO_2_ concentration in vago‐sino‐aortic denervated rats (Moreira et al. 2006). These findings strengthen the idea that the small increase in CO_2_/H^+^ induced by airway obstruction might activates RTN neurons and result in an increase in PNA and sympathoexcitation in intact and CBD rats. Furthermore, the difference between PNA and SNA responses induced by airway obstruction during hyperoxia in chemo intact and CBD rats suggest a potential interaction between peripheral chemoreceptors and central chemoreceptors.

### The role of NTS neurotransmission in PNA, SNA, and cardiovascular responses induced by airway obstruction

The primary site of afferent inputs from peripheral chemoreceptors is the NTS. In addition, NTS also integrates input from baroreceptors, pulmonary receptors, and cardiopulmonary receptors. Prior studies suggest that glutamatergic neurotransmission mediates reflex responses to peripheral chemoreceptors activation (Vardhan et al. 1993; Sapru 1996), to baroreceptors activation (Guyenet et al. 1987; Gordon and Leone 1991) and to pulmonary stretch receptors activation (Bonham and McCrimmon 1990; Miyazaki et al. 1999). Since obstructive apnea may activate all of afferents input, we tested whether interruption of NTS neurotransmission would attenuate airway obstruction‐induced responses. Inhibition of NTS neurons or blockade of NMDA/non‐NMDA glutamatergic receptors reduced PNA, SNA, and cardiovascular responses during upper airway obstruction. In addition, both treatments drastically attenuated these same responses during chemoreflex activation and the sympathoinhibition by baroreflex activation thereby confirming the effectiveness of the approach. Interestingly, airway obstruction produced a biphasic response in ABP. This response could be attributed to numerous factors as discussed above. Although animals were breathing 100% O_2_, a small reduction in arterial oxygen saturation was observed (~6%) thereby raising the possibility that hypoxia might contribute. Future studies are needed to investigate this possibility. Altogether, these findings indicate that part of airway obstruction responses depend on glutamatergic NTS neurotransmission.

A recent study demonstrated that the blockade of NTS neurotransmission by muscimol drastically reduced respiratory and splanchnic SNA response to hypoxia (8–10%) in anesthetized rats (Favero et al. 2011). In contrast, microinjection of muscimol did not modify respiratory and splanchnic SNA response to 10% of CO_2_ (Favero et al. 2011). Favero et al. (2011) applied the same volume and concentration of this study and the injection was done into commissural NTS. Altogether, these results reinforce the hypothesis that central mechanisms contribute to the cardiovascular, respiratory, and SNA responses mediated by airway obstruction.

### Summary and Conclusion

The current findings demonstrate that 20 sec of upper airway obstruction evokes changes in PNA and SNA that partly depend on hypoxia, peripheral chemoreceptors afferents, and NTS neurotransmission. The partial attenuation achieved by any of these manipulations suggests that additional mechanisms (i.e., central chemoreceptors and/or central respiratory networks) may be involved in the cardiorespiratory and SNA changes induced by airway obstruction. These findings are important since the majority of studies use intermittent hypoxia stimulus to investigate the mechanisms involved in OSA. Here, we showed that airway obstructive is a complex stimulus that involves activation and integration of several mechanisms. In addition, our findings indicate that future studies aimed to investigate the pathophysiology of OSA should consider multiple approaches or models to better understand cardiorespiratory physiology.

## Conflict of Interest

None.
